# Potential Effects on Travelers’ Air Pollution Exposure and Associated Mortality Estimated for a Mode Shift from Car to Bicycle Commuting

**DOI:** 10.3390/ijerph17207635

**Published:** 2020-10-20

**Authors:** Johan Nilsson Sommar, Christer Johansson, Boel Lövenheim, Anders Markstedt, Magnus Strömgren, Bertil Forsberg

**Affiliations:** 1 Department of Public Health and Clinical Medicine, Section of Sustainable Health, Umeå University, 901 87 Umeå, Sweden; bertil.forsberg@umu.se; 2 Department of Environmental Science, Stockholm University, 106 91 Stockholm, Sweden; Christer.Johansson@aces.su.se; 3 Environment and Health Administration, SLB, 104 20 Stockholm, Sweden; boel.lovenheim@slb.nu; 4 WSP Civils, 121 88 Stockholm, Sweden; anders.markstedt@WSPGroup.se; 5 Department of Geography, Umeå University, 901 87 Umeå, Sweden; magnus.stromgren@umu.se

**Keywords:** air pollution, vehicle emissions, bicycle, bicyclist exposure, human health

## Abstract

This study aims to use dispersion-modeled concentrations of nitrogen oxides (NOx) and black carbon (BC) to estimate bicyclist exposures along a network of roads and bicycle paths. Such modeling was also performed in a scenario with increased bicycling. Accumulated concentrations between home and work were thereafter calculated for both bicyclists and drivers of cars. A transport model was used to estimate traffic volumes and current commuting preferences in Stockholm County. The study used individuals’ home and work addresses, their age, sex, and an empirical model estimate of their expected physical capacity in order to establish realistic bicycle travel distances. If car commuters with estimated physical capacity to bicycle to their workplace within 30 min changed their mode of transport to bicycle, >110,000 additional bicyclists would be achieved. Time-weighted mean concentrations along paths were, among current bicyclists, reduced from 25.8 to 24.2 μg/m^3^ for NOx and 1.14 to 1.08 μg/m^3^ for BC. Among the additional bicyclists, the yearly mean NOx dose from commuting increased from 0.08 to 1.03 μg/m^3^. This would be expected to yearly cause 0.10 fewer deaths for current bicycling levels and 1.7 more deaths for additional bicycling. This increased air pollution impact is much smaller than the decrease in the total population.

## 1. Introduction

High exposures to air pollutants occur both inside vehicles due to the proximity of air intakes to exhaust emissions from neighboring vehicles as well as while walking or biking alongside roads [1]. Commuting by bicycle during rush hour along densely trafficked corridors may contribute a substantial fraction (20–30%) of the total daily exposure (e.g., Hanninen et al. (2004) [2] and Dons et al. (2012) [1]). In Stockholm, measurements by diffusive NO_2_ samplers showed that the average concentration for a bicycle commute was higher than five times the urban background concentration [3].

A review with a meta-analysis including seven studies estimated that the air pollution concentrations (particles of size less than 2.5 μm (PM2.5)) along the route taken by bicyclists was 17% lower compared with the route taken by motorists [4]. Bicycling, however, increases both intake and uptake of air pollutants as a result of an increased ventilation rate, depth of respiration and oral breathing. A review of urban bicyclists’ exposures found that the minute ventilation was between 2 and 4.7 times higher compared to when driving a car, when bicycling between 12 and 24 km/h [5]. A review and meta-analysis of inhaled doses found that although the median exposure was 18% lower among active commuters compared with car drivers, the inhaled dose of pollutants was 4.5 times higher [6]. Daigle et al. (2003) [7] assessed the deposition of ultrafine particles (polydisperse carbonaceous ultrafine aerosol particles) in healthy human subjects at rest and during exercise. They found that the ventilation during bicycling with a moderate intensity increased from 11.5 to 38.1 L/min, corresponding to a ratio of 3.3. In addition, the uptake, the fraction of the particle mass that is deposited, increased by 31% from 0.58 to 0.76.

The health impact related to air pollution exposure when bicycling has been estimated within both studies evaluating the benefits of public bicycle sharing systems [8,9,10] and studies evaluating scenarios of increased bicycling (e.g., de Hartog et al. 2010 [11]; Rojas-Rueda et al. 2011 [9]; Woodcock et al. 2013 [10]). Air pollution exposure during bicycling was estimated based on air pollution concentrations, bicycling time, intake and uptake. In these studies, different assumptions regarding increased intake and uptake were made, varying between two and eight times as high among bicyclists compared to drivers of cars. Studies estimating exposure differences between bicycling and driving a car have done this based on measurement studies. This provides estimated average concentrations as a bicyclist and as a driver of a car, but to our knowledge, no previous study has estimated accumulative concentrations along actual individual trips. Such individual estimates can also consider individual differences in bicycling speed (e.g., considering age, gender and distance), affecting the accumulated air pollution exposure.

The aim of this study is to estimate nitrogen oxide (NOx) and black carbon (BC) concentrations along bicycle paths among current bicycle commuters, the expected change in these exposures among potential bicyclists that previously commuted by car and the resulting impacts on mortality in a scenario of increased bicycle commuting.

## 2. Materials and Methods

### 2.1. Defining Current and Alternative Modes of Commuting

#### 2.1.1. Current Modes of Travel

Travel survey data were used to obtain an estimate of the proportion currently traveling to work with each mode of transport; walking, bicycling, public transport and car [12]. These proportions of travel modes were estimated between all pairs of small statistical areas (home and work) within Stockholm County (population 2.3 million), where the size of each area depends on the population density, but also by considering natural divisions of neighborhoods. These data were incorporated into the LuTrans model together with data on traffic flows on roads [13]. The model is regularly calibrated based on traffic counts and the travel output is modeled as a logit model of: (1) travel survey data allocating individual journeys to different modes of transport and (2) traffic counts to allocate car trips to specific car routes. The output is traffic flow on each link in the model, where a link is defined as the connection between two major intersections in the road network. In the present form, there are auto links and public transport links included in the model.

Using this model, a current mode of transport to and from work was allocated to individuals with a home and work address within Stockholm County. Individual information on age, gender, car ownership and home and work address was obtained from the ASTRID database [14].

#### 2.1.2. Mode-Shift Scenario

The individual data on home and work address, age and gender contained in the ASTRID database were also used to identify individuals that, according to a model predicting bicycling speed, have the potential to bicycle to work within 30 min.

The coordinates for the home and work address were extracted and the shortest path along a network of possible roads and bicycle paths was determined. If the individual was estimated to have the potential to bicycle to work within 30 min based on age and gender, and the individual was previously allocated as traveling to work by car, the individual in the mode-shift scenario switched to travel by bicycle.

The individual expected bicycle speed was based on a sample of about 455 existing male and female bicycle commuters within the same population of Greater Stockholm, Sweden. These participants were all recruited through advertisements in newspapers, and all the details of the recruitment and the sample characteristics have been described by Schantz (2017) [15]. The participants drew their own normal bicycle commuting route to work on a map, and its distance was measured using a criterion method [16]. The bicycling duration was self-reported and was requested in the questionnaire to represent the duration of a normal day without any errands on the way. These analyses have been described in detail by Schantz (2017) [15]. This sample of individuals may, however, not be representative of the cycling speed in the general population. Therefore, expected bicycling speeds were scaled down according to the relative difference in maximum oxygen uptake comparing current bicycle commuters and a sample from the general population. This was conducted separately within age and gender groups. Since these two samples of individuals were taken at different time points, a scaling was also performed according to gender-specific time trends in BMI within the population. This scaling of bicycle speed to the general population has been described in detail by Schantz et al. (2018) [17], where the resulting bicycle speeds were given by

speed (km/h) = 0.719 ∗ (34.8 + 0.31 ∗ age) among men(1)

and

speed (km/h) = 0.763 ∗ (25.9 + 0.21 ∗ age) among women,(2)

where age was measured in years and 0.719 and 0.763 represent the bicycle commuter to the general population effect among men and women, respectively.

The LuTrans traffic model was used to model traffic flows in the mode-shift scenario where car trips had been transferred to bicycle. A demand matrix was used to estimate the route for each car trip, and since the demand within the road system decreased due to a reduced number of cars, a new traffic flow was estimated, where remaining traffic may choose a different route. Each road was defined by short road links and the result of such a change was at the road link level, which thereafter was used to calculate vehicle emissions and traffic pollution concentrations.

In order to calculate air pollution exposures among bicycle commuters, it is necessary to know where the bicycle network is separated from the road network. For this purpose, the bicycle network was imported from digital data provided by the national road database (NVDB) [18] and the local road databases (LVDB) [19].

### 2.2. Vehicle Emissions

The emission inventory for Greater Stockholm includes some 40,000 road links and an annual traffic volume of 12,000 million vehicle kilometers [19,20]. NOx, particulate matter (PM) exhaust and BC emissions from road traffic are described with emission factors (emission per km driven). Vehicles are grouped into passenger cars (petrol and diesel), light commercial vehicles, heavy goods vehicles and buses. Emission factors of NOx and PM exhaust for different vehicle types, speeds and driving conditions were calculated based on HBEFA 3.2 [21]. BC emission factors were based on the Transphorm project [22].

### 2.3. Dispersion and Exposure Modeling

The concentrations and exposures with and without the mode-shift scenario were compared using the same meteorological conditions, i.e., only changing the emissions. The concentrations due to local road traffic emissions were calculated using a wind model and a Gaussian air quality dispersion model, both part of the Airviro Air Quality Management System (http://www.airviro.com). The system has been used in Stockholm for more than 20 years and it has provided exposure estimates for several epidemiological studies and health impact assessments [23,24,25].

Meteorological input for the dispersion model was based on a climatology created from 15 years of meteorological measurements (15 min averages) from a 50 m high mast located in the southern part of Stockholm. The climatology consists of a list of hourly events, each with a certain frequency of occurrence, which together yielded a distribution of different weather conditions that was similar to the distribution of the full scenario period (for further details, see Johansson et al. (2007) [24]). The dispersion calculations were performed with a 25 square meter resolution describing the average diurnal variation in concentrations along different roads depending on the traffic volume, composition and atmospheric dispersion. The effects of buildings on the dispersion were considered using a street canyon model, OSPM, also part of the Airviro system. The concentrations alongside streets were used to estimate the exposure dose for people bicycling instead of driving a car.

The results from the dispersion calculations were adjusted to represent weekdays in April to October (excluding July) during morning rush hour between 7 a.m. and 9 a.m. Each road and bicycle link got an individual weighted average concentration from the adjusted concentration grid using the intersect tool in a GIS program.

The accumulated air pollution concentration along the routes taken as a bicyclist and as a motorist, respectively, was calculated as the sum of the link concentration (*C_i_*) times the time spent on that link (*t_i_*):

(3) $$\sum_{i=1}^{n}C_{i}t_{i},$$

where *i* = (1, 2, …, *n*) is the link sequence between home and work.

Doses of NOx and BC were calculated assuming four round trips a week, 45 weeks a year. Time not spent commuting was assumed to be spent at home. Population weighted exposures at home addresses were retrieved from Johansson et al. (2017) [26]. The change in ambient concentrations, population exposure and the impact on mortality among citizens have been reported without any analysis of in-traffic exposure [26].

### 2.4. Estimation of Dose among Bicyclists and Motorists

In epidemiological studies of long-term exposure to ambient air pollutants and adverse health effects in adults, e.g., increase in mortality, exposure is measured or modeled as the outdoor concentration. For air pollutants judged to be important for the health effects, such as fine particles and nitrogen dioxide, the infiltration rate into buildings is typically around 40–60% [27,28]. The infiltration into car cabins is of the same magnitude [29,30], but depends on a number of factors including the vehicle ventilation, filter efficiency and particle size distribution [31,32]. Based on these results, we have assumed that pollutant concentrations are reduced by 50% inside the car cabin compared to outside. Since the participants in the epidemiological studies could be assumed to have spent 90–95 percent of the time indoors, their actual exposure to the studied outdoor pollutants should be about half of the estimated outdoor concentration. Thus, the true increase in relative risk per actual increase in exposure dose is likely about twice as large per unit as the outdoor exposure–response relation indicates. For this reason, we approximately double the relative risk for the dose from bicycling.

Due to higher ventilation rate, bicycling leads to a higher intake of pollutants, around three-fold for PM2.5 and five-fold for ultrafine particles [5]. Based on the study by Daigle et al. (2003) [7], we assumed the minute ventilation to be 230% (3.3 times) higher for bicyclists compared with being at rest indoors or sitting in a car, and that uptake was increased by 31%, resulting in an increased dose of 430%.

Based on these assumptions about bicyclists’ and motorists’ exposures, we calculated (1) the contribution to the yearly mean exposure as a result of the increased air pollution dose for the individuals that previously drove a car to work but instead start to bicycle, and (2) the reduction of the yearly mean among current bicycle commuters. Since the travel time between home and work may differ as a bicyclist compared to as a motorist, the mean exposure at the home address was used for the time not spent commuting.

### 2.5. Health Impact Calculations

The epidemiological studies with a finer spatial resolution which can capture the gradients in exposure to local traffic pollutants indicate an important effect of local traffic emissions, resulting in high relative risks [33,34]. Of particular interest is a Norwegian study of 16,000 men from Oslo, of whom 25% died during the follow up, which used modeled nitrogen oxides (NOx) in the residential area as the exposure indicator [35]. This cohort, with people between 40–49 years of age at the start of the study, was followed from 1972/73 through to 1998. NOx was estimated in a model with 1000 m grids, and a street contribution was added for the largest streets. When the median concentration of NOx for 1974-78 was used, the relative risk for total non-violent mortality was 1.08 per 10 µg/m^3^ (95% CI 1.06–1.11).

Additionally, for black carbon (BC), cohort studies have provided sufficient evidence of associations between all-cause and cardiopulmonary mortality with long-term average BC exposure. Studies of short-term health effects show that the associations with BC are more robust than those with PM2.5 or PM10, suggesting that BC is a better indicator of harmful particulate substances from combustion sources (especially traffic) than undifferentiated PM mass. For BC, we used a pooled estimate for premature mortality associated with long-term exposure to elemental carbon of 6% per 1 μg/m^3^ (95% CI 5–7%), as reported in a review by Hoek et al. (2013) [36].

The baseline mortality in 2013 for individuals older than 30 years of age in the county of Stockholm was 1124 per 100,000 [37]. Age- and sex-specific mortality was used to estimate the number of preterm deaths and life years lost/gained due to an individual potential change in air pollution exposure.

The subpopulation of Stockholm County that had their home and work address within the county consisted of 923,970 individuals. The mean age was 41.8 years and 49% were women.

All calculations were made using the statistical software R [38].

## 3. Results

### 3.1. Effects on Mode of Commuting

In the current scenario, 6% traveled by bicycle between home and the workplace, 38% drove a car, 4% as a passenger of a car, 38% by public transport and 14% walked (Table 1). In the mode-shift scenario, i.e., with 111,000 switching from a car to bicycling, 18% would travel by bicycle and 26% would drive a car.

**Figure 1:**
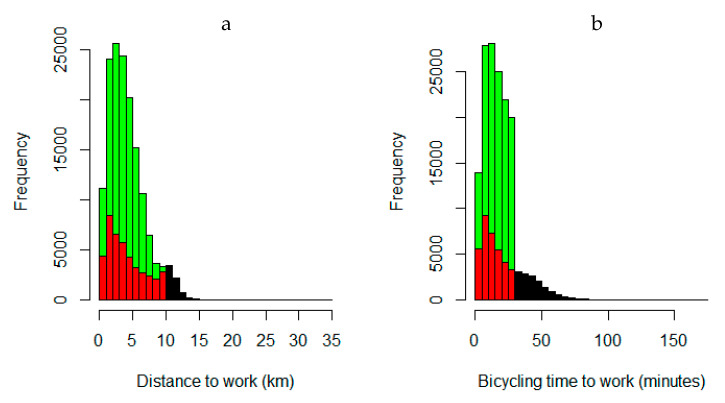
(**a**) Distribution of bicycle distances to work. (**b**) Distribution of travel times to work by bicycle.

Median travel distance to work was 3.5 km among current bicyclists compared to 3.3 km among new bicyclists in the mode-shift scenario. Distributions of travel distances to work within scenarios are presented in Figure 1a. Corresponding travel time distributions in scenarios are presented in Figure 1b, where mean travel times were 22 min and 18 min, respectively. The mean age among new cyclists was 42 years and 48% were women.

**Figure 2:**
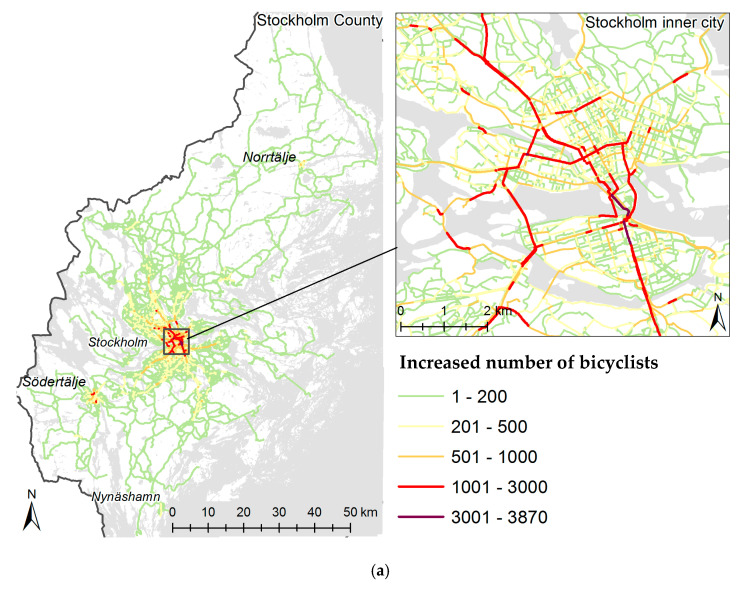

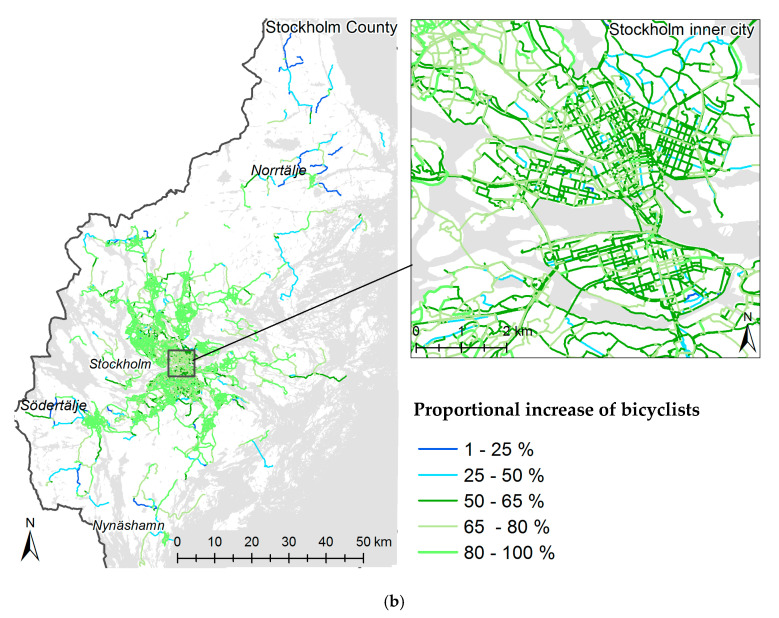
(**a**) Number of new bicyclists along different roads in the county of Stockholm (left) and in the inner city of Stockholm (right). (**b**) Proportional increase in the number of bicyclists along different roads in the county of Stockholm (left) and in the inner city of Stockholm (right).

The geographical distribution of increased bicycling is presented in Figure 2a as the additional number of bicyclists on each part of the road network within the whole county and the inner city of Stockholm. The largest increase occurred in the inner city, where the number of bicyclists increased by up to 3870 per day on some parts of the road network. Corresponding proportional decreases are presented in Figure 2b.

### 3.2. Effects on NOx and BC (Black Carbon) Concentrations

#### 3.2.1. NOx

**Figure 3:**
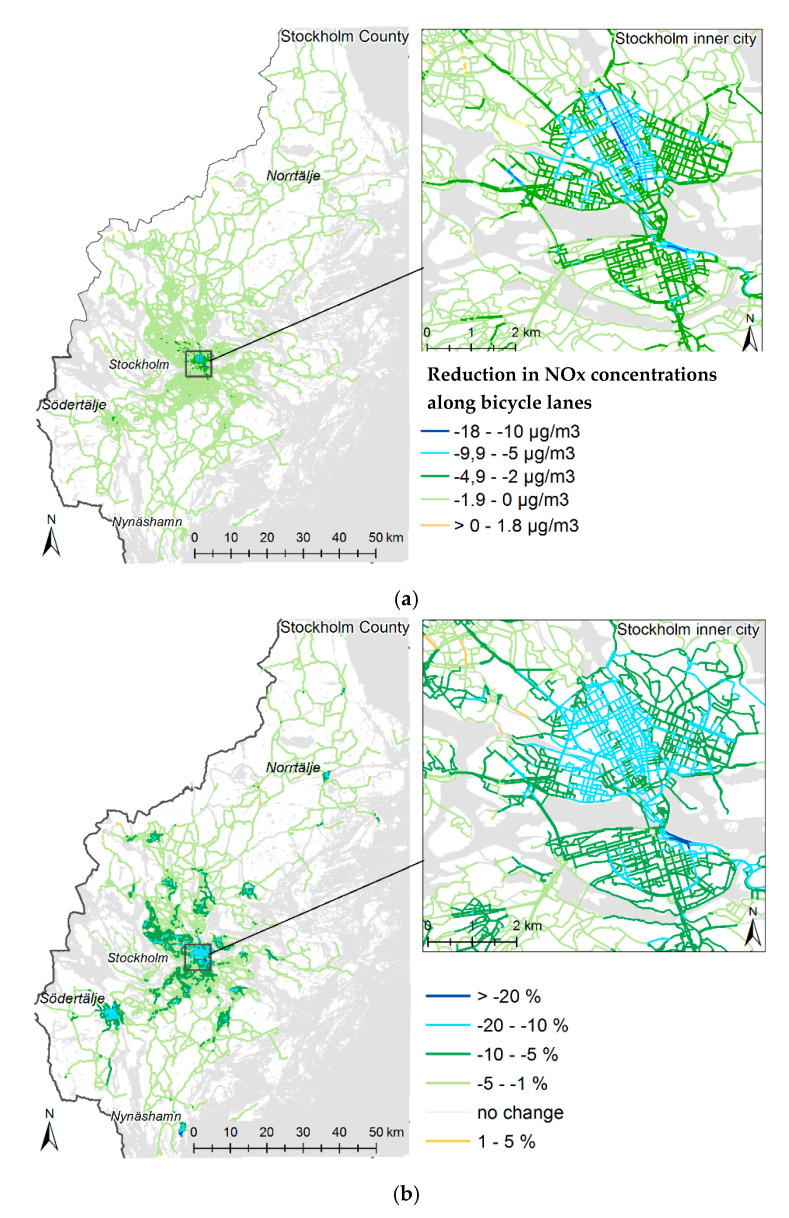
(**a**) Reduction in nitrogen oxide (NOx) concentrations along roads in the county and inner city of Stockholm when the 30 min scenario is realized compared to the current situation. (**b**) Proportional reduction in NOx concentrations along roads in the county and inner city of Stockholm when the 30 min scenario is realized compared to the current situation.

Up to a 13.8 μg/m^3^ reduction in traffic contribution to NOx concentrations was calculated along the road network, as shown in Figure 3a. This corresponds to up to a 21% reduction in the traffic contribution to NOx concentrations (Figure 3b). The mean reduction in concentration for the whole area was 6%, with the largest reductions occurring in the most densely populated areas. A higher mean NOx concentration was found along roads available for driving compared with bicycle paths before the mode-shift, 23.2 vs. 16.6 µg/m^3^.

The average cumulative concentration for a one-way trip between home and work among current bicycle commuters was currently 9.7 μg/m^3^ * h. In the mode-shift scenario, the average cumulative concentration was reduced to 9.1 μg/m^3^ * h (6% reduction). Among the individuals that changed mode of transport from car to bicycle, the mean cumulative concentration for drivers of cars was currently 3.8 μg/m^3^ * h and within the mode-shift scenario as bicyclists 5.8 μg/m^3^ * h (53% higher).

Corresponding time-weighted mean concentrations along paths were, among current bicyclists, 25.8 μg/m^3^ and in the mode-shift scenario, 24.2 μg/m^3^ (6% lower). Among the individuals that changed their mode of transport from car to bicycle time-weighted concentrations were 15.0 μg/m^3^ as motorists in the current scenario and 21.1 μg/m^3^ as bicyclists in the mode-shift scenario (41% higher).

#### 3.2.2. BC

The mean BC concentration was 24% higher along roads available for driving compared with along bicycle paths, 0.72 vs. 0.58 μg/m^3^. Time-weighted mean concentrations of BC among current bicyclists were 1.14 μg/m^3^ and 1.08 μg/m^3^ within the current situation and mode-shift scenario, respectively (a reduction by 5%). For the new bicyclists, these time-weighted mean concentrations were 0.75 and 0.96 μg/m^3^ (28% higher after the mode shift).

### 3.3. Effects on NOx and BC Doses

#### 3.3.1. NOx

**Figure 4:**
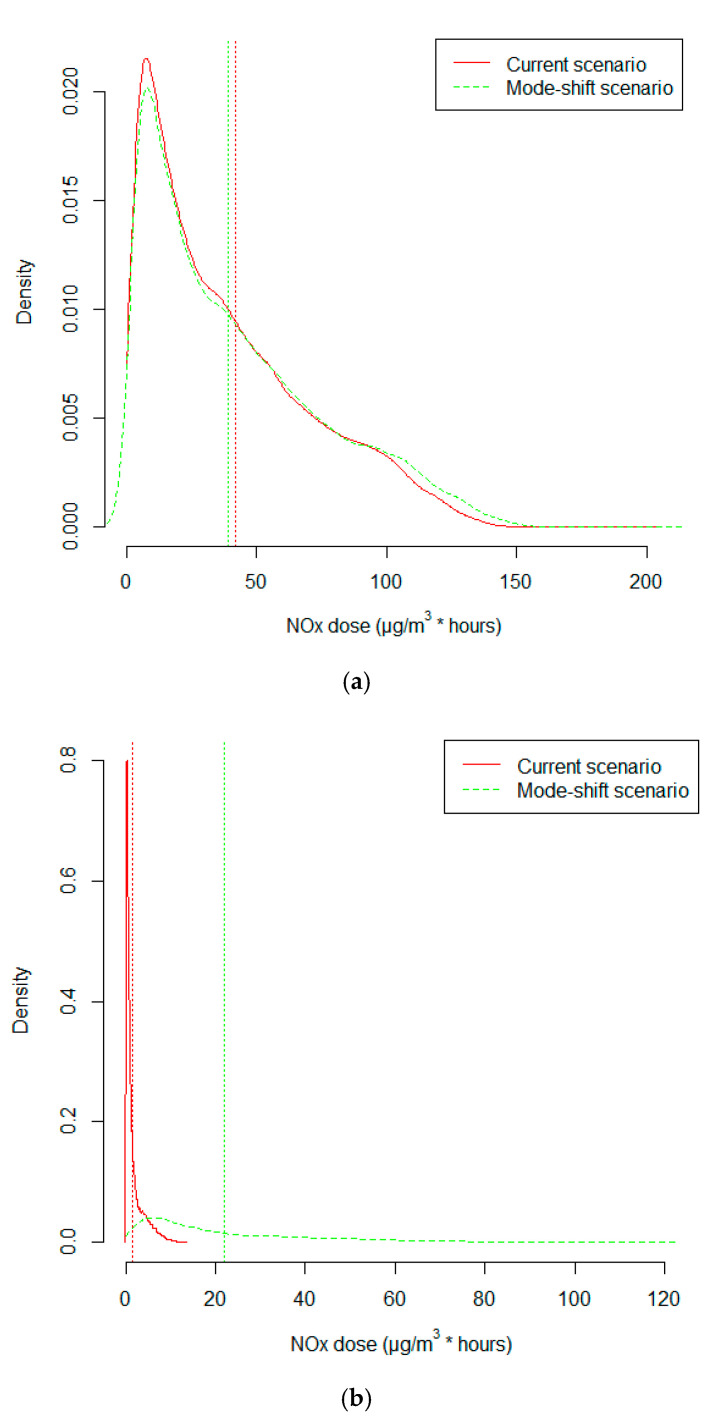
(**a**) Distribution of cumulative NOx doses for a one-way trip between home and work among current bicyclists. (**b**) Cumulative NOx dose for a one-way trip between home and work among the individuals that changed their mode of transport from car to bicycle. To contrast between the two modes of transport, the difference in travel time was resolved by assuming that time not spent commuting was spent at home.

Assuming an average increased intake and uptake of 330% among bicyclists due to the increased ventilation rate, the mean cumulative NOx dose among current bicyclists was reduced by 6%, from 41.8 to 39.1 μg/m^3^ * h (Figure 4a). By further assuming a 50% reduced NOx concentration inside the car due to air filtering, the corresponding one-way mean cumulative dose between home and work among the new bicyclists was 1.9 μg/m^3^ * h as a driver of a car and 25.1 μg/m^3^ * h as a bicyclist (a 13-fold higher dose; Figure 4b).

Among current bicyclists, the mean NOx dose weighted according to travel times was 111 μg/m^3^ in the current transport scenario but decreased to 104 μg/m^3^ in the mode-shift scenario. The weighted mean doses among new bicyclists were 7.5 μg/m^3^ as a driver of a car and 90.6 μg/m^3^ as a bicyclist.

On average, the yearly mean NOx dose decreased by 0.11 μg/m^3^ among current bicycle commuters when comparing the current situation with the mode-shift scenario. The contribution to the yearly mean by the bicycle commute decreased from 1.71 to 1.61 μg/m^3^. Among the individuals that changed their commuting mode of transport from car to bicycle, the yearly mean NOx dose from commuting changed from 0.08 to 1.03 μg/m^3^, an increase of 0.95 μg/m^3^.

#### 3.3.2. BC

The mean BC doses weighted according to travel times among current bicyclists were 4.92 and 4.66 μg/m^3^ in the current situation and mode-shift scenario, respectively (5% lower). Weighted concentrations among the individuals that changed mode of transport to bicycling were 0.37 and 4.11 μg/m^3^ (11 times higher). Corresponding contributions to the yearly mean changed from 0.08 to 0.07 μg/m^3^ among current bicyclists and 0.004 to 0.05 μg/m^3^ among the individuals that changed their mode of transport to bicycling.

### 3.4. Impact on Mortality Related to Changed NOx and BC Exposure among Bicyclists

#### 3.4.1. NOx

These changes in yearly mean NOx exposures among current bicycle commuters would be expected to decrease yearly mortality from the current 64.6 deaths to 64.5 deaths (a decrease by 1%), corresponding to 1735 and 1732 years of life lost, respectively. The mortality thus decreased by 0.10 deaths corresponding to 2.63 life years.

Among the new bicycle commuters, the increased NOx exposure during bicycle commuting compared to driving a car would be expected to cause 1.70 yearly preterm deaths, corresponding to 48.0 years of life lost. Current number of yearly deaths was 133.4 with 3672 years of life lost compared with 135.1 deaths and 3720 years of life lost with increased NOx exposure (a 1% increase).

#### 3.4.2. BC

Expected impacts on mortality due to the estimated reduction in BC exposure among current bicyclists corresponded to 0.02 preterm deaths and 0.59 years of life lost (a reduction by 0.03%). Among the individuals that changed their mode of transport to bicycling, the increased BC exposure would be expected to increase the number of yearly preterm deaths by 0.48 (0.4%), corresponding to 13.4 years of life lost.

### 3.5. Counterfactual Exposures

NOx and BC exposures were also calculated for the current bicycle commuters if they instead would have driven a car. This was performed based on dispersion-modeled NOx and BC concentrations within the current traffic scenario, i.e., the same amount of traffic. Time-weighted NOx and BC concentrations along the route taken as a driver of a car were 18.2 and 0.87 μg/m^3^, respectively. Corresponding doses were 9.1 and 0.44 μg/m^3^, contributing 0.13 and 0.006 μg/m^3^ to the yearly mean.

## 4. Discussion

This is the first study that estimate bicyclists’ and motorists’ air pollution exposure in a population using actual trips based on register data on individuals’ home and work address. The time-weighted mean NOx dose currently in Stockholm County was estimated to be 72% higher among bicyclists compared with those currently driving a car. The difference in exposure was due to an increased ventilation rate and uptake of air pollutants when bicycling, a lower air pollution concentration inside cars compared to outside and the choice of route. The study also assessed the health impact of a change in air pollution exposure due to a shift from driving a car to bicycling, in a scenario were current drivers of a car would shift to a bicycle if they had the capacity to bicycle to their work place within 30 min. This scenario assessment showed a significant effect on preterm mortality for the individuals that changed their mode of transport to bicycling, but only minor effects on current bicycle commuters. Within previous health impact assessment studies, such scenarios have been created by, e.g., transferring some proportion of commuters to bicycling, without information about which routes would be replaced. For the general population, this scenario has been estimated to reduce all-cause mortality by 69 deaths per year, resulting in more than 449 years of life saved annually for the Stockholm County area with 2.1 million inhabitants [26].

A quantitative review has been performed on the ratio between air pollution concentrations along routes as a bicyclist compared with a driver of a car [4]. The review included European measurement studies with a simultaneous or quasi-simultaneous design. For particles with a diameter less than 2.5 μm (PM2.5), they found that the mean concentration along the route taken as a driver of a car was 20% higher than that taken as a bicyclist. Results were, however, varying. Black carbon (BC) concentrations were on average 70% higher and CO 90% higher, whereas concentrations of ultra-fine particles (UFPs) were similar. Results for BC and CO were, however, only based on two studies. A difficulty with synthesizing exposures is that bicyclists and cars share roads to varying degrees in different places. Our dispersion-modeled result for the current traffic situation had, on average, 31% higher time-weighted BC concentrations along routes taken as a bicyclist compared with routes taken as a driver of a car. Measurements of personal exposure to traffic-related air pollution by diffusive NO_2_ samplers in Stockholm showed that the average concentration per route among active commuters (walking or cycling) ranged from 48 to 105 µg NO_2_/m^3^, which was more than five times the urban background [3]. This contributed 16.4 µg NO_2_/m^3^ to the annual mean exposure.

Estimating the air pollution exposure as a driver of a car requires knowledge about air filtration in cars. In-vehicle ultrafine particulate matter concentrations, compared to outside concentrations, have been found to be 15% lower with the window down, 47% lower with the windows closed but the ventilation open and 83–90% lower with windows and ventilation closed [29]. The result is in agreement with a measurement study comparing particle mass concentrations when walking with concentrations inside a car, as the time-averaged fine particle (PM2.5-PM1) mass concentration was found to be 2.2 times higher for the individual walking compared to the individual driving [30]. For fine particle (PM1) mass concentrations, the factor was 1.9. However, for coarse particle (PM10–PM2.5) mass concentrations, the factor was found to be 4.7.

Ventilations rates when bicycling have been measured in several studies. In a review, it was found that bicycling between 8 and 24 km/h increased the ventilation by between 80 and 390% compared to a driver of a car [5]. The ventilation ranged between 22 and 59 L/min among bicyclists. Besides differences in bicycle speed, the range of ventilation would also be influenced by the terrain, the bicycle weight and condition, weather and the bicyclist’s fitness. In the current study, the individual bicycle speed was estimated based on age and gender using data among current bicycle commuters within Stockholm. Since the knowledge about age- and gender-specific ventilation rates for bicyclists are limited, we used a common average ventilation rate to calculate the exposure dose. A portion of the inhaled air pollutant gases are absorbed, and particles deposited on the lining of the respiratory tract or pass through to the bloodstream. The air pollution uptake dose is the proportion of inhaled pollutants that remain in the body. A part of the absorbed air pollution gases and deposited air pollution particles will be further transported and reach other body tissues, but a part will also be expelled through mucociliary clearance or desorption. Daigle et al. (2003) [7] found that when subjects increased their ventilation from 11.5 to 38.1 L/min, the proportion of particles that was not exhaled increased from 0.66 to 0.83 and the particle mass not exhaled from 0.58 to 0.76. Therefore, the 230% higher ventilation increased the total mass deposition by 330%.

In previous health impact assessments (HIAs) of increased bicycling, different assumptions have been made about intake and uptake of air pollutants during physical activity. A recent review about air pollution as a risk factor in HIAs of a travel mode shift towards cycling found that assumed ventilation rates ranged between two and eight times higher among bicyclists compared to drivers of cars [39]. Two HIAs assumed ventilation 2.2 times higher based on two measurement studies from the Netherlands [40,41]. The increase in respiration has been based on such measurement studies but also on physical activity intensities. The metabolic rate during bicycling is then compared to the metabolic rate during rest. Individual specific metabolic rates are often assumed to be 3.5 mL O_2_/min/kg body mass. To ease comparisons between metabolic rates, the metabolic equivalent of task (MET) is used. MET values are calculated as the ratio between individual metabolic rates. The “Compendium of Physical Activities” [42] lists bicycling “to/from work, self-selected pace” as 6.8 METs and “leisure” bicycling at 5.5 mph as 3.5 METs. In a study using accelerometer measurements, a 41 L/min ventilation rate corresponded to 6.58 METs [43]. Such MET values have in some studies been used to estimate bicyclists’ respiration to obtain air pollution dose assessments. These differences in intensity and ventilation affect the air pollution dose and thus expected health impacts.

In a review with air pollution dose estimates, including a meta-analysis, comparing air pollution doses among bicyclists with drivers of cars and found that the median dose was 4.5 (with first and third quartiles of 3.3 and 6.7) times higher among bicyclists [6]. The finding was mainly due to a higher ventilation rate and depth of breathing among bicyclists. Active commuters, and particularly pedestrians, also have a longer exposure time.

In a systematic review, 17 HIA studies of a change in mode of transport from car to bicycle were reviewed [44]. Seven of the studies estimated the impact of a change in air pollution exposure among travelers, of which four also estimated the impact of a change in air pollution exposure within the general population. All seven studies reported an increase in the average air pollution dose among individuals that changed their mode of transport from car to bicycle. Woodcock et al. (2013) [45] estimated the average route exposure to PM2.5 as a bicyclist and as a driver of a car to be 15.75 μg/m^3^ and 17.80 μg/m^3^, respectively. However, considering differences in ventilation and position on the road, the dose of PM2.5 was 6.8 times the average exposure as a bicyclist and 1.95 times the average exposure as a driver of a car, resulting in, on average, a more than three times higher dose as a bicyclist compared to a driver of a car. In a study from the Netherlands, mean concentrations during 30 min of bicycling and driving a car were estimated to be 34.5 μg/m^3^ and 40.0 μg/m^3^, respectively [11]. The inhaled dose per day as a bicyclist was reported to be on average 22.8 μg/m^3^ compared to 12 μg/m^3^ per day as a driver of a car. De Hartog et al. (2010) [11] also estimated commuting concentrations of black smoke for a 30 min commute, reporting a mean bicycle concentration of 18.2 μg/m^3^ and a mean concentration while driving a car of 30.0 μg/m^3^. Our results on time-weighted mean BC concentrations along routes were 1.14 and 0.87 μg/m^3^ as a bicyclist and driver of a car, respectively. Incorporating the benefit of increased physical activity, but increased accident risk, Woodcock et al. (2013) [45] also found that the impact of increased exposure to air pollution only corresponded to about 1% of the total impact and de Hartog et al. (2010) [11] found less than 10%. Such risk–benefit analyses were also conducted by Tainio et al. (2016) [46], where the risk associated with bicycling in areas with a 100 μg/m^3^ PM2.5 concentration was estimated to outweigh the benefit of physical activity after 1.5 h. If instead the counterfactual was driving a car, the long-term benefit of physical activity would exceed the risk during up to 3.5 h of bicycling. A HIA of restricting active commuting on days with high levels of air pollution (above 150 μg/m^3^ PM2.5), where commuters instead would use public transport, found that this would not reduce all-cause mortality in any of the six cities (Helsinki, London, Sao Paulo, Warsaw, Beijing, New Delhi) that were studied [47].

These previous HIAs have assessed air pollution impacts among travelers based on PM2.5 concentrations. Hartog et al. (2010) [11], however, also assessed health impacts in relation to black smoke. In that study, a relatively large health impact related to air pollution was obtained, compared to studies assessing PM2.5 concentrations. Part of the reason is the PM2.5 exposure response function for mortality used in the other studies. This relative risk of 1.06 per 10 μg/m^3^ has mainly been derived from multiregional analyses of large cohorts. Since these regions had different PM2.5 background concentrations, the relative risk, to a large degree, reflects differences in secondary (non-local) particle matter background concentrations from urban monitoring (e.g., Hoek et al. (2013) [36]). Our study assessed health impacts by changes in NOx and BC, both highly correlated with vehicle exhaust toxic constituents. A high correlation has been found between NOx and total particle concentrations in Stockholm, both at curb-side sites [24,48] and close to the highway [49]. Epidemiological studies with high-resolution air pollution concentrations able to capture the gradients in exposure to local traffic pollutants have found high levels of associated health risks [33,34]. A meta-analysis of cohort studies on the long-term effect of NO_2_ on total and natural mortality found for Europe a 6.6% (95% CI 2.9–10.4%) increased risk per 10 μg/m^3^ [50]. The review also presented a compilation of multipollutant results of NO_2_ effects adjusted for PM2.5 concentrations (not BC), where associations with NO_2_ to a large extent remained after adjustment. Even though the correlations between NO_2_ and PM2.5 were between 0.7 and 0.8, and effects were thus possibly confounded, an independent effect of NO_2_ on mortality was also suggested by a higher interquartile range relative risk for NO_2_ compared to PM2.5. Therefore, HIAs based on only PM2.5 concentrations may neglect important sources of the air pollution mixture, while adding impacts estimated for NO_2_ and BC may cause some double counting.

The policy implication of our findings, also considering the findings from previous studies, may be that, from a health perspective, it is important that expansions of bicycling infrastructure consider the location with regard to air pollution exposure. The results further highlight the importance of air pollution mitigation in urban environments.

As in all risk assessments, our study was limited by the availability of data and the necessity of making assumptions. The bike paths contain stops; however, bicyclists were assumed to maintain a constant speed. Since concentrations of air pollutants may be higher in these traffic intersections than elsewhere on the bicycle route, exposures may be somewhat underestimated. Similarly, even though drivers of a car could take a different route due to less traffic congestion within the scenario with increased bicycling, we were not able to incorporate such congestion when estimating the cumulative air pollution exposure among drivers. Instead, the exposure was estimated assuming flowing traffic. Individual data on mode of transport were not available and therefore imputation of mode of transport within areas was performed according to travel survey information and information on car ownership. Using air pollution risk estimates from elsewhere involves uncertainty because air pollution components are location and source specific [51]. A similar relative risk estimate used for NOx from Nafstad et al. (2004) [35] has also been found been found in a Swedish cohort only slightly older at the start of follow up than participants in the study from Oslo [35]. More studies are available on the long-term effect of NO_2_, and the relative risks for NOx are in agreement with many of these studies.

## 5. Conclusions

Reducing air pollution exposure affects mortality among bicycle commuters, and there is thus a potential to reduce preterm mortality among bicyclists by reducing vehicle exhaust and road wear. Transferring car commuters to bicycles would, however, considerably increase those individuals’ air pollution dose, resulting in a non-negligible air pollution effect on mortality. Further research on ventilation among bicyclists at different levels of exertion, as well as intake and uptake of air pollutants, is warranted. The yearly number of deaths due to air pollution exposure is estimated to increase by 1.7 among the additional bicyclists. However, for the total population, this mode-shift would lower air pollution exposure and has been estimated to avoid 69 deaths per year [26].

## Figures and Tables

**Table 1:** Frequencies and proportions of the individuals utilizing different types of transport.

	Current Situation		Mode-Shift Scenario		Difference and Proportional Change	
Mode of Transport	Number of Individuals	Proportion	Number of Individuals	Proportion	Number of Individuals	Proportion
Bicycling	53,206	6%	164,693	18%	111,487	210%
Walking	130,441	14%	130,441	14%	0	0%
Public transport	352,412	38%	352,412	38%	0	0%
Car (driver)	352,614	38%	241,127	26%	−111,487	−32%
Car (passenger)	35,297	4%	35,297	4%	0	0%
